# Dr. P. A. Allaire, of Aurora, Ill.

**Published:** 1885-10

**Authors:** M. M. Robbins, C. B. Slater

**Affiliations:** President; Secretary


### OBITUARY.

The Aurora Medical Society, at a regular meeting, indicated the feeling of the society in the following expression: Again are we reminded that we are mortal. Death indeed loves a shining mark; our ranks have again been invaded by its ruthless presence, and a second time, within the space of a few weeks, we are called upon to follow to his last resting place another of our members, Mr. Pierre A. Allaire, one to whom we were wont to look for counsel; one whose mental training had been such as to give him a preeminent position in the ranks of his profession; one who despised shams; an honest man. Such were the qualities we found in our association with our deceased brother. While we sincerely mourn his loss, his example will ever prove an incentive to greater effort on our part to qualify ourselves for success in the profession of our choice. Our hearts go out in sorrow toward his bereaved family, and we hereby tender them our warmest sympathy.

M. M. ROBBINS, President.
C. B. SLATER, Secretary.

*September 18, 1885.*

